# Supplementary material for: Exon junction complex proteins bind nascent transcripts independently of pre-mRNA splicing in Drosophila melanogaster
Source: eLife. 2016 Nov 23;5:e19881. doi: 10.7554/eLife.19881 (PMC5158136; doi:10.7554/eLife.19881)
Supplement: Supplementary file 1. — Sequence of primers for real-time RT-PCR or amplification of dsDNA fragment for in-vitro transcription of dsRNAs used for RNAi. Labels refer to gene names. DOI: http://dx.doi.org/10.7554/eLife.19881.020 [file elife-19881-supp1.doc]

**Supplementary file 1.** List of primers used

| **Primer** | **Sequence** |
| --- | --- |
| Y14 (F) | 5’-ATTGACAATGCGGAGGAGTTCGAGG-3’ |
| Y14 (R) | 5’-CGGTGCTTCGCCTTTTCCTTCAG-3’ |
| MAGO (F) | 5’-CGTGATGGAAGAACTGAAGCGAATCA-3’ |
| MAGO (R) | 5’-TCGAGGTGGTGAACGAGATGTGC-3’ |
| eIF4AII (F) | 5’-GAGCGAGGATGTGGAGGTGA-3’ |
| eIF4AII (R) | 5’-CGTAATGCTCCTTTGCTGAATGG-3’ |
| RpL 32 (F) | 5’-CGCCGCTTCAAGGGACAGTAT-3’ |
| RpL 32 (R) | 5’-TCTTGAGAACGCAGGCGACCG-3’ |
| 18s RNA (F) | 5’-ACCGGTGGAGTTCTTATATGTGAT-3’ |
| 18s RNA (R) | 5’-CGGCCCACAATAACACTCGT-3’ |
| NCM (F) | 5'-GAGCGGAGGAAGGATGAAGAGCAG-3' |
| NCM (R) | 5'-CGGTAGGTCATCTTCGTCTTTGTCG-3' |
| Y14 RNAi (F) | 5’-T7-CGATGTGTTGGACATTGACA-3’ |
| Y14 RNAi (R) | 5’-T7-GACGCTTTTCGGACTTTTT-3’ |
| MAGO RNAi (F) | 5’-T7-CACGGAGGACTTTTACCTAC-3’ |
| MAGO RNAi (R) | 5’-T7-ATATGGGCTTGATCTTGAAATG-3’ |
| eIF4AIII RNAi (F) | 5’-T7-GACGAATTGACACTGGAAGG-3’ |
| eIF4AIII RNAi (R) | 5’-T7-AGAATATTAGTTTAGATCAAGTCAG-3’ |
